# The Neat Dance of COVID-19: NEAT1, DANCR, and Co-Modulated Cholinergic RNAs Link to Inflammation

**DOI:** 10.3389/fimmu.2020.590870

**Published:** 2020-10-09

**Authors:** Chanan Meydan, Nimrod Madrer, Hermona Soreq

**Affiliations:** ^1^Department of Internal Medicine, Mayanei Hayeshua Medical Center, Bnei Brak, Israel; ^2^Sackler School of Medicine, Tel Aviv University, Tel Aviv, Israel; ^3^Central District, Leumit Health Services, Tel Aviv, Israel; ^4^The Department of Biological Chemistry and The Edmond and Lilly Safra Center for Brain Sciences, The Alexander Silberman Institute of Life Sciences, The Hebrew University of Jerusalem, Jerusalem, Israel

**Keywords:** cholinergic, central nervous system, COVID-19, long non-coding RNA, miRNA

## Abstract

The COVID-19 pandemic exerts inflammation-related parasympathetic complications and post-infection manifestations with major inter-individual variability. To seek the corresponding transcriptomic origins for the impact of COVID-19 infection and its aftermath consequences, we sought the relevance of long and short non-coding RNAs (ncRNAs) for susceptibility to COVID-19 infection. We selected inflammation-prone men and women of diverse ages among the cohort of Genome Tissue expression (GTEx) by mining RNA-seq datasets from their lung, and blood tissues, followed by quantitative qRT-PCR, bioinformatics-based network analyses and thorough statistics compared to brain cell culture and infection tests with COVID-19 and H1N1 viruses. In lung tissues from 57 inflammation-prone, but not other GTEx donors, we discovered sharp declines of the lung pathology-associated ncRNA DANCR and the nuclear paraspeckles forming neuroprotective ncRNA NEAT1. Accompanying increases in the acetylcholine-regulating transcripts capable of controlling inflammation co-appeared in SARS-CoV-2 infected but not H1N1 influenza infected lung cells. The lung cells-characteristic DANCR and NEAT1 association with inflammation-controlling transcripts could not be observed in blood cells, weakened with age and presented sex-dependent links in GTEx lung RNA-seq dataset. Supporting active involvement in the inflammatory risks accompanying COVID-19, DANCR’s decline associated with decrease of the COVID-19-related cellular transcript ACE2 and with sex-related increases in coding transcripts potentiating acetylcholine signaling. Furthermore, transcription factors (TFs) in lung, brain and cultured infected cells created networks with the candidate transcripts, indicating tissue-specific expression patterns. Supporting links of post-infection inflammatory and cognitive damages with cholinergic mal-functioning, man and woman-originated cultured cholinergic neurons presented differentiation-related increases of DANCR and NEAT1 targeting microRNAs. Briefly, changes in ncRNAs and TFs from inflammation-prone human lung tissues, SARS-CoV-2-infected lung cells and man and woman-derived differentiated cholinergic neurons reflected the inflammatory pathobiology related to COVID-19. By shifting ncRNA differences into comparative diagnostic and therapeutic profiles, our RNA-sequencing based Resource can identify ncRNA regulating candidates for COVID-19 and its associated immediate and predicted long-term inflammation and neurological complications, and sex-related therapeutics thereof. Our findings encourage diagnostics of involved tissue, and further investigation of NEAT1-inducing statins and anti-cholinergic medications in the COVID-19 context.

**Graphical Abstract:**
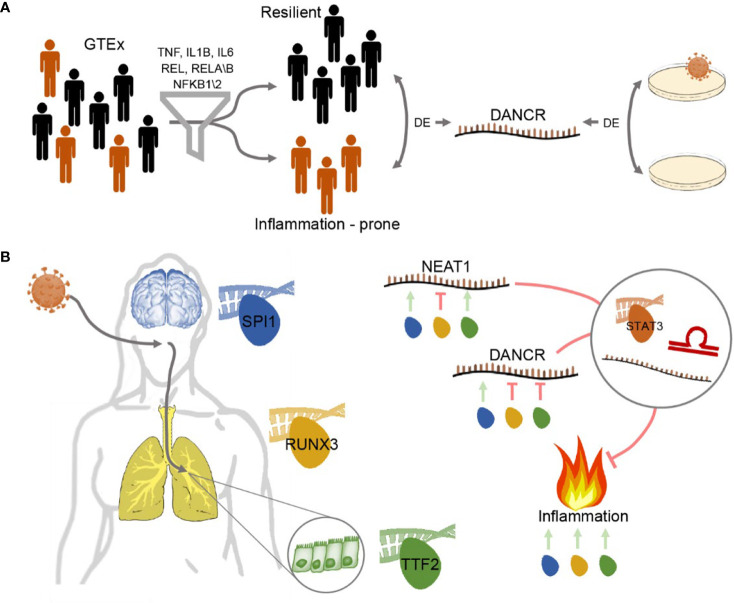
Study workflow - DANCR and NEAT1 interact with inflammatory markers in a sex- and tissue-specific manner **(A)** Segregating blood and lung samples from the GTEx dataset of healthy post-mortem donors into resilient and inflammation prone. Samples expressing high levels of seven of the inflammation biomarkers IL-1B, IL-6, NFKB1\2, REL, RELA\B were defined as inflammation-prone, and others as resilient. Differential expression analysis highlighted DANCR as a lncRNA DE between the two groups. Parallel analysis of cultured lung epithelial cells infected with SARS-CoV-2 found DANCR’s decline in infected cells. **(B)** Both GTEx post-mortem men and women lung and brain tissues and lung-derived cell culture infected with SARS-CoV-2 showed DANCR and NEAT1 changes in inflammatory biomarkers, part of which show modified levels under COVID-19 infection. Three different TF controllers of DANCR and NEAT1 can mediate the inflammatory tone in diverse organs (SPI1 (blue) in cortex, RUNX3 (yellow) in lung and TTF2 (green) in lung epithelial cells). DANCR and NEAT1 can block inflammation *via* interacting with other ncRNAs, sponging miRs, or affecting TFs like STAT3. Red, flat-headed and green arrows indicate expression blockade and induction of expression, respectively.

## Introduction

The COVID-19 pandemic presents an ongoing challenge to the medical and research community. SARS-CoV-2 is estimated to have infected globally more than 25 million people and claimed the lives of at least 800,000 by August 30th, 2020 (World Health Organization COVID-19 Weekly Epidemiological Update, retrieved September 5th, 2020). The clinical natural history of COVID-19, generated by SARS-COV-2, includes 4–6 days of incubation time, followed by a febrile and respiratory-focused clinical picture, and occasionally complicated by superinfection and multiple organ failure. While the majority of cases are mild with eventual resolution, or not reaching a clinical threshold for seeking care (1), country-specific preliminary data indicates a highly variable death rate. To date, treatment of COVID-19 is mainly supportive, mostly addressing pulmonary and systemic decompensation, and involves experimental medical approaches including inflammation modulators (2–8). Age and chronic medical conditions are the most common COVID-19-related adverse prognostic factors, including cardiovascular-spectrum diseases and cancer. However, predicting who is at risk for severe complications is challenging, as accumulating follow-up data is still insufficient (9–11), and the realm of delayed cognitive risks has not yet been addressed.

Clinical and imaging reports indicate that COVID-19 elicits massive inflammatory events, with acute respiratory distress syndrome (ARDS) evident in about 20%–30% of hospitalized patients (12, 13), and cardiac complications ascribed to inflammation (14, 15). ARDS is classically associated with high mortality, and COVID-19 patients with ARDS present substantial associated mortality of approximately 50% (4, 16). ARDS is also a key feature of other related viral diseases [e.g. Severe Acute Respiratory Syndrome (SARS) and Middle-East Respiratory Syndrome (MERS)] (4). Highly active in ARDS are pro-inflammatory mediators such as inducible Nitric Oxide Synthase (iNOS), tumor necrosis factor (TNF) and interleukin-1β (IL-1β), leading to organ-related detrimental effects in lung and vasculature (17–19), including thrombotic events (20, 21). COVID-19-induced lung disease also associates with hyper-inflammatory profile, reflecting its potential for developing ARDS and other inflammation-related complications (3).

A variety of neurological COVID-19 sequelae have been reported and described in preliminary studies so far. These include encephalitis, characterized by irritability, confusion and reduced consciousness coupled with signs of brain inflammation; entities involving demyelination (myelitis and encephalomyelitis); encephalopathy without brain inflammation, characterized by changed personality, behavior, cognition or consciousness; seizures; polyradiculopathy; and cerebrovascular manifestations (22–26). More commonly, in a European study of 417 COVID-19 patients, circa 85% of mild to moderate patients reported smell and taste sensory alterations (27). Ergo, neurological involvement of COVID-19 is not limited only to patients surviving complicated courses, with cerebral gross inflammation/infarction, mechanical ventilation, or intensive care, but also occurs in milder cases without such adverse features.

We predicted that COVID-19 may exert its effects *via* modifying gene expression in the infected organs. These changes would involve both protein-coding genes and non-coding RNAs (ncRNAs), which consist of the great majority of human RNAs and many of which are multi-leveled regulators of cellular function, including inflammation (28–31). NcRNAs span short microRNAs (miRs), long non-coding RNA (lncRNAs) longer than 200 nucleotides, and other types of ncRNAs. Under acute or chronic challenges, ncRNAs interact with other ncRNAs, coding messenger RNAs (mRNAs), or directly with proteins, modifying their actions and affecting various clinical conditions including inflammation (32, 33). Specifically, several ncRNAs react to chronic, subacute inflammation, such as in the metabolic syndrome and its components and in the organismal response to infections (34, 35). Therefore, impaired ncRNA reactions in clinical scenarios may include inflammation-induced “collateral damage”. In silico analysis shows that SARS-CoV-2 may be targeted by over 800 human microRNAs (miRs); circa 65% of which target the related SARS-CoV, but over 300 miRs are unique in comparison. This change in the affected ncRNA network may lead to the different clinical manifestations and infectivity which are apparent in the current pandemic (36). Since lncRNAs form a complex infection-associated activity profile and may operate as “sponges” modulating miR regulatory activities and response to infection (37–39), we expected changes in the levels of such ncRNAs to relate to the pathogenesis and hyper-inflammatory nature of COVID-19 (12, 40) as well as to its predicted risk of neurologic sequelae (24, 41–43).

Autonomic imbalance, favoring sympathetic activation, is frequently evident in ARDS models. Given that cholinergic signaling can affect both immediate and long-lasting body and brain reactions (44, 45), we expected lncRNAs to contribute to the surveillance over cholinergic signaling in response to COVID-19. Neuroimaging follow-up of ARDS survivors further highlights mental sequelae, as well as accelerated cerebral and hippocampal atrophy (18). Correspondingly, neurological complications are evident for COVID-19, in ambulatory, inpatient and intensive care settings (24, 43). These include ischemic cerebrovascular infarctions, intracranial hemorrhage, encephalopathy, and other neuropathological patterns (46). The closely related SARS-CoV has also been associated with neuro-invasiveness (47), and neurological complications are well recognized in other infectious and post-infectious scenarios (48), as are the associated elements of inflammation and ncRNAs (49–51). To address this concept, we sought the potential roles of differentially expressed (DE) ncRNAs in the Genome Tissue Expression (GTEx) RNA-seq datasets from lung, brain and blood of healthy men and women across ages, analyzed RNA-seq datasets of SARS-CoV-2 infected lung cells and experimentally tested the expression patterns of miRs and lncRNAs in human-originated neuronal cell cultures of man and woman origin under cholinergic differentiation.

## Materials and Methods

### Human Cell and Tissue Analyses

To address the issue of individual diversity of transcript changes in COVID-19, we selected a representative subset of inflammation-related transcripts including IL-1β, IL-6, TNFα, NFkB1\2, REL, and RELA\B (52), and divided GTEx project datasets (https://www.gtexportal.org/) of 563 lung and blood tissues from apparently healthy individuals to those originated from “non-inflammation-prone” and “inflammation-prone” individuals. In 57 samples out of those 563, the lung tissues expressed at least seven out of the listed inflammation-related transcripts above the tissue median. Based on that, we classified these samples as “inflammation-prone”.

### RNA-Seq Bioinformatics

To identify short and long RNAs whose levels change under SARS-CoV-2 infection and under neuronal cholinergic differentiation, we used DESeq2 (53), essentially as recently reported (31). Further, we mined RNA-sequencing datasets of cultured lung epithelial cells from lung adenocarcinoma patients exposed to SARS-CoV-2 (Multiplicity of Infection, MOI=0.2) and human bronchial epithelial cells (MOI=2), both sequenced 24 h post-infection (54); we also compared our findings in SARS-CoV-2 infected cells to a dataset of H1N1 (associated with the “swine flu” pandemic) infected human bronchial epithelial cells, compared to non-infected control cells (55), at 24 h post-infection. We likewise mined short RNA-sequencing datasets from the Calu-3 epithelial lung cells infected with SARS-CoV-2 (MOI=0.3) as compared to mock-infected cells, analyzed differentially expressed (DE) miRs accounting for time post-infection, and compared these changes to those of DE miRs whose levels change under neuronal cholinergic differentiation in human-originated neuroblastoma cells from male and female donors (LA-N-5\2; n=4 biological replicates for each condition). DE miRs were then integrated into a network of interactions using the miRNet-2.0 online tool (https://www.mirnet.ca/). In parallel, we examined long RNAs (coding and non-coding) from Calu-3-infected cells at different post-infection timepoints. Lastly, we analyzed datasets from neuron progenitor cells (SUNE-1) in which DANCR was knocked down using siRNA compared to control cells treated with scramble transcripts.

### Experimental Validation

To challenge the relevance of the identified DE transcripts to the features of cholinergic brain neurons, we subjected cultured female-originated neuroblastoma cells (LA-N-2, passage 6) to cholinergic differentiation, by 4 days of exposure to 440_pM_ of CNTF (56). Cells were grown in EMEM (M5650, Sigma Aldrich), F-12 (N4888, Sigma Aldrich), FSC, L-glu and PSA in a ratio of 1:1:0.5:0.01:0.01. Following 4 days of CNTF treatment, cells were harvested (washed with 1 volume of PBS (P4417, Sigma Aldrich) and then treated with QIAzol (QIAGEN). We extracted RNA and compared the changes in cholinergic and lncRNA transcript levels by qPCR (SYBR^®^ Green PCR Master Mix) and normalized the results to the GAPDH housekeeping gene (qPCR, n=3 biological replicates for each condition). All cell lines used were of passage 9 or below.

**Table 1** presents a list of all the cellular and organismal systems which were analyzed throughout our study and their corresponding details. Ethical approval for these experiments was confirmed by the Hebrew University’s Committee for research involving human-derived materials.

**Table 1:** The analyzed datasets.

Cell\Tissue	Source	Treatment	Evaluation method	Dataset	Reference	Figs
NHBE	Human epithelial cells	SARS-CoV-2 \ mock infection	long RNA sequencing	GSE147507	Blanco-Melo et.al. (54)	1,2
A549	Lung adenocarcinoma	SARS-CoV-2 \ mock infection	long RNA sequencing	GSE147507	Blanco-Melo et.al. (54)	1
NHBE	Human epithelial lung cells	H1N1 \ mock infection	long RNA sequencing	GSE48575	Paquette et.al. (55)	2
LA-N-2	Female neuroblastoma	CNTF \ PBS treatment (see *Experimental validation* for protocol elaboration)	qPCR	Own experimental work	—	3
LA-N-2	Female neuroblastoma	CNTF \ PBS treatment	short RNA sequencing	GSE132951	Lobentanzer et.al. (56)	6
LA-N-5	Male neuroblastoma	CNTF \ PBS treatment	short RNA sequencing	GSE132951	Lobentanzer et.al. (56)	6
Calu-3	Human epithelial lung cells	SARS-CoV-2 \ mock infection	short and long RNA sequencing	GSE148729	Wyler et.al., 2020 (preprint (57))	4,6
SUNE-1	Neuronal progenitor cells	siDANCR \ siSCR transfection	long RNA sequencing	GSE117415	Wen et.al. (58)	6
Post-mortem samples	human lung, brain and blood	none	long RNA sequencing	GTEx	—	1,3,4,5

Listed are the RNA-seq datasets which were analyzed throughout this study, the originating cell types, tissues, and treatments thereof, the experimental and analysis tools used to produce and mine these datasets and the figures where the outcome results of these analyses are presented.

### Statistics

The statistical analysis in this study was conducted using R studio, multiple comparisons were corrected using FDR. Boxplots represent t-tests or ANOVAs and point plots represent Pearson correlation tests. Networks of interactions were conducted using the online tool miRNet 2.0. All of the RNA-sequencing data was CMP normalized, and upon comparison of two different datasets, was z-score normalized as well (i.e. the mean expression of each gene was reduced from each count and the result was divided by the gene’s standard deviation, yielding a dataset in which the mean expression of every gene is 0 and its standard deviation is 1). Upon running a DESeq analysis we accounted for batch effect if existence of such effect was suspected.

## Results

### Lung Tissues With Inflammatory Profile Show Altered DANCR Levels

**Figure 1:**
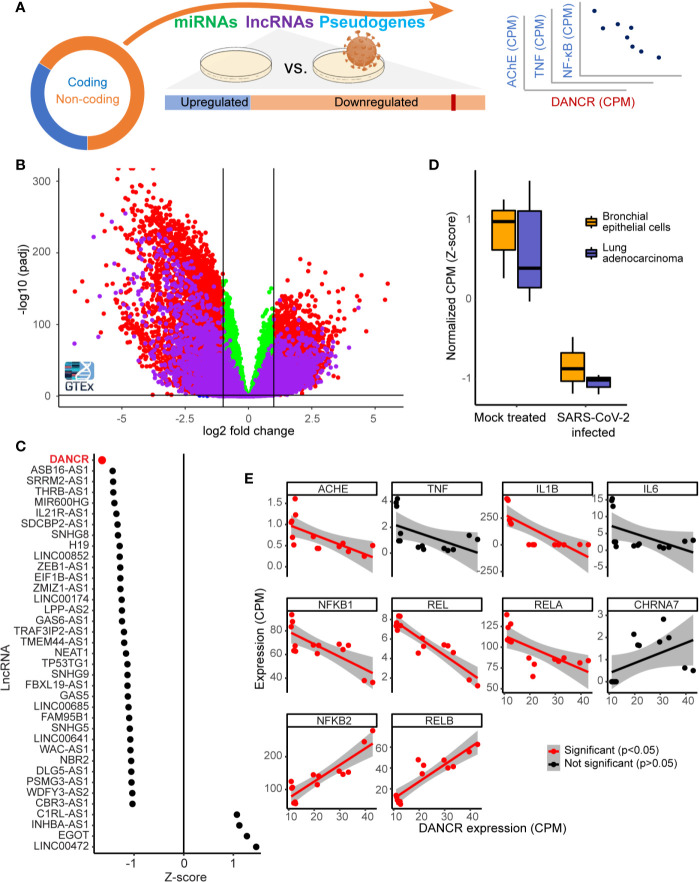
DANCR is downregulated in SARS-CoV-2 infected lung cells and in GTEx inflammation-prone lung samples. **(A)** The majority of the transcriptome (circle) consists of non-coding-RNA, including miRs, lncRNAs, and other RNAs. Changes in lncRNAs were assessed in viral-infected lung cell lines and inflammation-prone GTEx donors, and the expression levels of the down-regulated lncRNA DANCR was compared to those of inflammation-related coding transcripts. **(B)** Volcano plot showing coding transcripts (red and green) and lncRNAs (purple) from lung and blood samples of healthy post-mortem donors (n=419 and 407, respectively) with or without inflammatory reactions. Horizontal line represents p = 0.05 and vertical lines indicate -1/1 log2 of fold change. **(C)** 69% of the lncRNAs expressed in NHBE or A549 lung cells were downregulated [shown are only those with p value <0.05; of those, only DANCR had adjusted p < 0.05 (FDR)]. **(D)** DANCR is downregulated under viral infection both in bronchial epithelial cells and in lung adenocarcinoma cells [NHBE, p < 0.013; A549, p < 0.008; t. test (FDR)]. **(E)** DANCR expression change was significantly correlated (red, p < 0.05, FDR) with those of the cholinergic-associated AChE, multiple cytokines and NFkB subunits in both SARS-CoV-2 infected and control lung cell lines.

Our working hypothesis predicted SARS-CoV-2 infection-induced changes in both lncRNAs and mRNAs coding for key inflammatory cytokines. To challenge this hypothesis, we mined RNA-seq datasets from 57 inflammation-prone GTEx individuals whose lung tissues expressed at least seven of the eight representative inflammation-related transcripts including IL-1β, IL-6, TNFα, NFkB1\2, REL, and RELA\B (52). These findings were compared to those of the 506 non-inflammation-prone healthy men and women at different age groups, including lung and blood datasets. Both lung and blood samples from the inflammation-prone individuals expressed large numbers of massively downregulated differentially expressed (DE) mRNAs and lncRNAs compared to the non-inflammation-prone samples (**Figures 1A, B**), potentially pointing at the transcriptomic interlinks connecting between the studied transcripts as relevant for the risk of infection. In particular, we noted DANCR, which showed a pronounced decline in the GTEx inflammation-prone lung tissues (p<0.1e-13, t.test). The 855 nucleotides long DANCR transcript is produced from chromosome 4q12 (59), and is known to modulate the action of CTNNB1 (catenin beta-1), which is actively involved in respiratory infections and sepsis (60–62). Previous studies show that DANCR can trigger the pro-inflammatory STAT3 activation *via* the IL-11-JAK2 pathway (63). STAT3, in turn, activates IL-1b, IL-6, NFkB1, REL, and RELB (64–66), as well as the transcription factor (TF) SPI-1 (Pu.1) which further interacts with TNF, NFkB2, RELA, DANCR, and the NEAT1-associated p54nrb paraspeckle protein (67–71).

To find lncRNAs downregulation which can be associated with SARS-CoV-2 infection in specific cell types, we mined RNA-seq datasets from SARS-CoV-2 infected compared to non-infected bronchial epithelial cells (NHBE) and lung adenocarcinoma cells (A549; GSE147507). Infection-related expression differences in these cells involved massive downregulation of numerous lncRNAs, with the levels of DANCR most prominently reduced (z-score of -1.65 compared to mock infected cells; p<0.01, FDR; **Figure 1C**) in infected cells of both cell lines (**Figure 1D**; NHBE, p<0.013; A549, p<0.008; t. test, FDR). Our current finding of DANCR’s decline in inflammation-prone lung tissue, in tandem with the accumulating evidence pertaining to DANCR’s role in inflammation and in infected cell cultures, led to our prediction that DANCR’s decline might play a role in COVID-19.

### The Decline in DANCR’s Expression Showed Inverse Correlations to Inflammation Controllers and Molecular Cholinergic Regulators

We next pursued correlation of DANCR’s expression levels in SARS-CoV-2 infected and control lung cells with those of IL-1β, IL-6, TNFα, and the five NFkB subunits, all established inflammation mediators. Since cholinergic signaling can block inflammation (72), we also examined the acetylcholine hydrolyzing enzyme acetylcholinesterase (AChE) and the inflammatory related α7 nicotinic receptor (CHRNA7). This analysis revealed negative and significant correlation between the levels of DANCR to transcripts composing the canonical pathway of inflammation-related NFkB activation, including REL, RELA, and NFkB1 and to AChE and IL-1β (**Figure 1E**; p<0.05, FDR, Pearson correlation test). In contradistinction, the levels of DANCR showed positive correlation to those of NFkB2 and RELB, which constitute the non-canonical NFkB pathway (73) that is downregulated in lung cells under influenza A infection (74) and is functionally involved in ARDS (73, 75). Altogether, this indicated an inverse cholinergic-related relationship between DANCR and inflammation, raising the possibility that DANCR’s decline is functionally relevant for the acute phase and/or the aftermath of such infections.

### COVID-19 and H1N1 Infection Show Distinct Inflammatory Signatures

**Figure 2:**
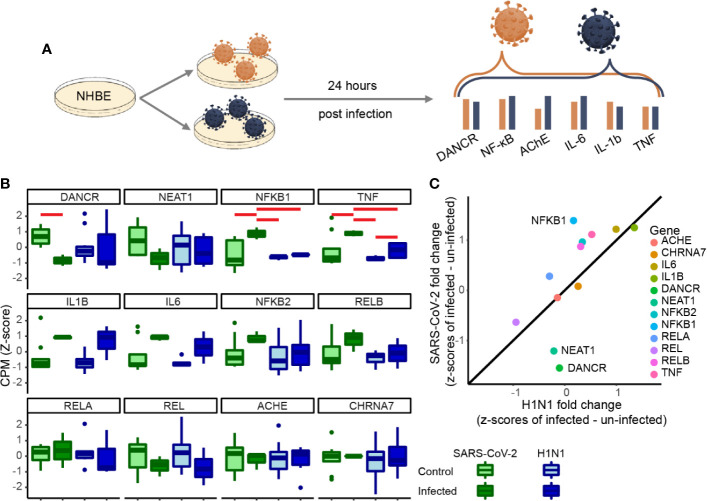
DANCR, NEAT1 and NFkB1 reactions differentiate between SARS-CoV-2 and pandemic H1N1 infection. **(A)** Normalized expression (CPM, z-score) of genes of interest in SARS-CoV-2\pandemic H1N1 [both 24 h post-infection (p. i.)] and control lung bronchial epithelial cells. **(B)** Boxplots show normalized expression of biological replicates (n = 3 for SARS-CoV-2 infected and control and for H1N1-infected and n=6 for H1N1 control). Red horizontal lines mark significantly different expression (p < 0.05 ANOVA, FDR). **(C)** Z-score fold changes of infected minus control SARS-CoV-2\H1N1. Black line shows y=x slope. NFkB1, DANCR, and NEAT1 show significant changes (p < 0.01), all other genes are found within the 0.99 SD of the slope.

To test if the changes we observed are unique to SARS-CoV-2 or are a general characteristic of respiratory infection, we examined the changes of the same gene set under pandemic H1N1 virus infection. For this purpose, we analyzed RNA-seq datasets from NHBE lung cells infected with either of these viruses 24 h post-infection. The inflammatory cytokines IL-1β and IL-6 as well as TNFα showed similar trends of increase in both infection protocols, albeit with more prominent increases of TNFα under SARS-CoV-2 infection (**Figures 2A, B**). Moreover, the inflammation regulator NFkB1 was only increased under SARS-CoV-2 infection (**Figure 2B**). Both DANCR and the sepsis-related neuroprotective lncRNA NEAT1, which initiates and maintains the membrane-less organelle of nuclear paraspeckles (30, 33, 39), were suppressed in SARS-CoV-2 infected cells (**Figure 2C**) (see corresponding Resource in **Supplementary Table 1** for the full list of lncRNAs changed in NHBE and A549 cells infected with SARS-CoV-2, in NHBE cells infected with pandemic H1N1, and in lung inflammatory samples). Together, these observations indicated that inter-related changes in DANCR, NEAT1, and NFkB1 might play specific regulatory roles in the natural history of SARS-CoV-2 infection.

### DANCR’s Inflammation Links Wear With Age

**Figure 3:**
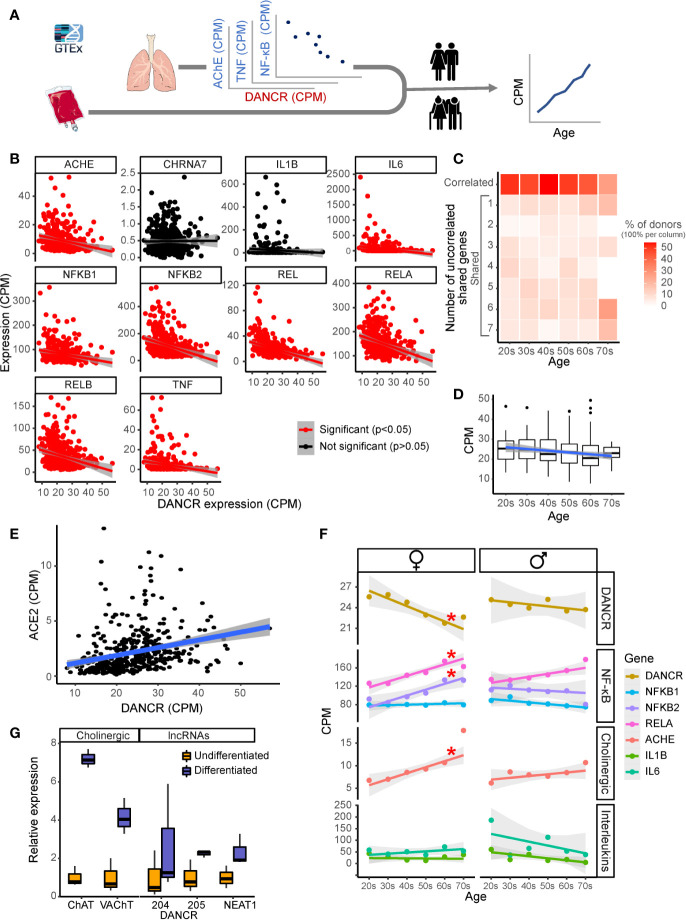
DANCR shows inter-related age- and sex-associated correlation with elevated lung inflammatory genes and associates with ACE2. **(A)** Expression of DANCR and other genes in 419 lung samples was followed post-mortem in healthy GTex individuals. Correlation between coding genes to DANCR expression was tested in lungs and age correlation—in lung and blood. **(B)** Significant (red, p < 0.05, FDR) and insignificant (black) correlation of DANCR to cholinergic- and inflammation-related genes in lung samples. **(C)** Heat-map showing age-related decline in DANCR’s correlation with the red-colored genes in B. The samples were sorted by the number of genes whose links were significant in the different age groups. Color scale shows calculated relative share of each subgroup of the total population at this age. A trend (R = 0.7, p < 0.1) was seen for the correlation of age with the fraction of donors whose values appeared significant. **(D)** Expression of DANCR in lung samples along age (R = -1.3, p < 0.008). Boxplots in grey show expression variability in each age group. **(E)** Correlated expression of ACE2 and DANCR in 419 lung samples from the GTEx dataset (R = 0.25, p < 0.0001, Pearson). **(F)** Correlation of various transcript levels with age in 427 lung samples. Red asterisks indicate significance of p < 0.05 (FDR). **(G)** Neuroblastoma cells of female (LA-N-2) and male (LA-N-5) origin were treated with CNTF to induce cholinergic differentiation, or PBS-treated for control cells. Four days post-treatment, the levels of lung transcripts were qPCR measured and normalized to GAPDH levels (n=3 biological replicates for each qPCR condition). ChAT and VAChT were upregulated in differentiated cholinergic neurons (p < 0.01, p < 0.05), the neuroprotective long variant of NEAT1 (p < 0.09) and two common transcripts of DANCR, DANCR-204 (709 bp) and DANCR-205 (748 bp) (p < 0.43, p < 0.063, respectively).

To obtain further predictive measures of the individual variability in the inflammation-related aspect of response to SARS-CoV-2 infection, and to assess DANCR’s link to this response at different ages (**Figure 3A**), we explored the association between DANCR expression levels and inflammation-related transcripts in 427 lung samples of GTEx donors. This screen showed direct correlation of DANCR’s expression levels to the expression of the inflammation-related genes, revealing similarly directed relationships to those observed in the infected cell lines (**Figure 3B**). Notably, up to 49% of the tested healthy individuals’ samples showed null association values between DANCR’s changes and those of each inflammation-related gene (meaning that the corresponding values appeared beneath the regression line), and 83% of those further presented null correlation to the change in DANCR for at least one of the selected inflammation-related genes, stressing pronounced inter-individual variability in these values. Moreover, estimating the share of the differently-reacting individuals in each age group noted a tendency for increase with age in the fraction of non-reacting individuals (**Figure 3C**; R=0.7, p<0.1), with parallel age-related decline of DANCR expression levels (**Figure 3D**; R=-0.13, p<0.008). We conclude that both the declined expression levels of DANCR and the gradual loss of DANCR’s association with the inflammation-related genes among GTEx donors could potentially reflect the loss of a protective process with age, among other age-related vulnerabilities to COVID-19.

Since SARS-CoV-2 infection depends on the expression of and interaction with the cellular ACE2 protein in host cells (76), we sought correlation between DANCR and ACE2 as well, and noticed a positive correlation between the expression of DANCR and ACE2 in lung samples from GTEx donors (**Figure 3E**; R=0.25, p<0.0001). This finding further supports the relevance of DANCR to the cellular response to SARS-CoV-2 infection. Inverse to the decline with age in DANCR’s expression levels, we noted a sex- and age-related increase in the lung expression levels of the acetylcholine hydrolyzing enzyme AChE as well as of the NFkB pathway members (**Figure 3F**), which was considerably stronger in females. Blood samples, in comparison, showed minimal age-related changes in DANCR expression levels (**Supplementary Figure S1**), highlighting the tissue specificity of this response. We conclude that the DANCR-related pathway is age- and sex-affected.

### DANCR and NEAT1 Affect Neural Tissue Through Cholinergic Mediators

The inflammatory association of the DANCR- and NEAT1-related networks and the inverse changes of DANCR and AChE along age predicted body-brain messages affecting the cholinergic network. Compatible with recent reports of cognitive damages in infected patients (23, 26), we considered that these interactions may reflect a response to the infection-induced inflammation, which might mitigate neuronal functioning and damage cognition during and after the acute phase. To experimentally search for changes in the cholinergic relationships of DANCR and NEAT1 within the nervous system, we subjected a female-originated neuroblastoma cell line (LA-N-2) to ciliary neurotrophic factor (CNTF)-induced cholinergic differentiation and compared the expression levels of both DANCR and NEAT1 before and after differentiation (**Figure 3G**). Successful acquirement of cholinergic properties was validated by measuring the expression levels of choline-acetyl transferase (ChAT) and the vesicular acetylcholine transporter (VAChT), both of which were significantly upregulated in differentiated cells (p<0.01, p<0.05; **Figure 3G**). Intriguingly, differentiated cholinergic neurons presented a selective increase of one of the splice variants of DANCR, DANCR-205 (748 bp; p<0.063), accompanied by upregulation of the nuclear paraspeckles-forming neuroprotective lncRNA NEAT1 (**Figure 3G**; p<0.09). Thus, both lung cells and brain neurons, but not blood cells, show informative changes in DANCR and its related inflammatory transcripts. Cholinergic neurons showed concomitant increases of expression of DANCR and NEAT1 with ChAT levels reflecting their acetylcholine producing capacities. As acetylcholine binding to the nicotinic acetylcholine receptor alpha7 can block NFkB and inflammation, we propose that DANCR and NEAT1 elevations may modify the inflammatory status of neurons.

### Transcription Factors Show Tissue-Specific Regulation of DANCR and NEAT1 Expression

**Figure 4:**
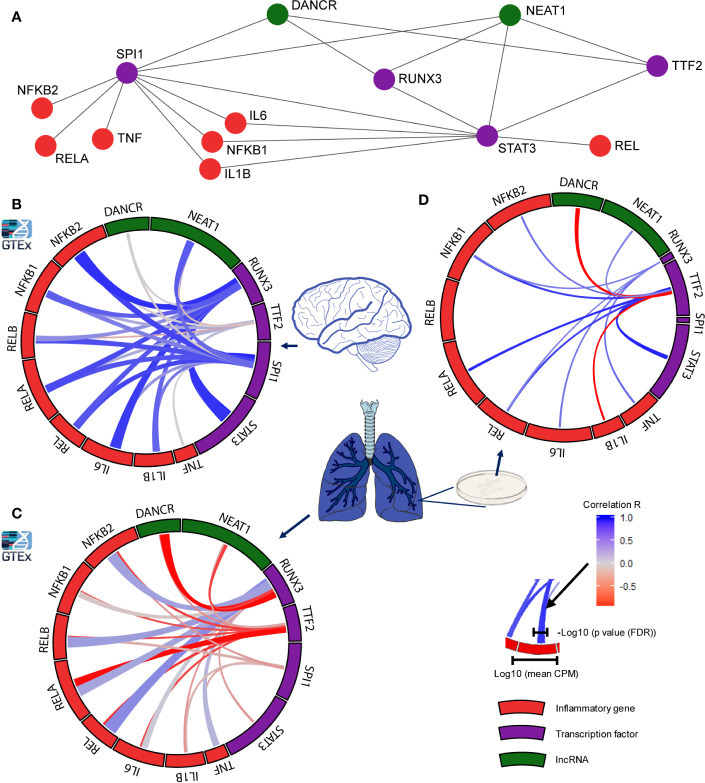
Distinct transcription factors affect the infection-related brain and lung impact of DANCR, NEAT1 and inflammatory transcripts. **(A)** Network presenting links of DANCR and NEAT1 (green) and inflammatory genes (red) with the transcription factors (TFs; purple) controlling their expression. **(B)** correlation between the expression levels of the various TFs to inflammatory (red) or lncRNA (green) transcripts in cortex samples from 158 post-mortem GTEx donors. The width of the bars on the surrounding circle represent log10 of the transcript’s CPM, the width of the tracks connecting the genes represents -log10 of the FDR corrected p value of the correlation between the two connected genes, and the color intensity of the track represents the correlation R (blue—positive and red—negative correlation). **(C)** As in B but for 427 lung samples. **(D)** As in B but for Calu-3 lung epithelial cell (from GSE148729; n=18).

Seeking mechanisms connecting DANCR’s expression with the upregulation of inflammatory biomarkers, we sought transcription factors (TFs) that co-regulate DANCR, NEAT1, and the inflammation-related genes. Mining the ENCODE dataset, we found three TFs targeting NEAT1 and DANCR: SPI1, RUNX3, and TTF2 (77, 78). Notably, RUNX3 and TTF2 can function both as enhancers and as suppressors of transcription (79–82). Beyond DANCR and NEAT1 these TFs can affect the transcription of STAT3, which operates as a TF modulator of inflammatory responses (83, 84) (**Figure 4A**). To assess the effect of these TFs as potential regulators of DANCR, NEAT1 and the inflammatory genes in lung and brain, we examined the global correlation of each of these TFs with genes/lncRNAs in 427 and 158 lung and cortex samples of GTEx donors, respectively. While cortical RUNX3 and SPI1 both showed strong positive correlation to DANCR, NEAT1 and the inflammatory genes (**Figure 4B**), lung SPI1 only showed weak correlation to the tested transcripts, and lung RUNX3 showed positive correlation to the inflammatory genes and negative correlation to DANCR and NEAT1 (**Figure 4C**). This matched the observation in NHBE cells where DANCR was negatively correlated with the inflammatory markers. RUNX3 is known to be activated upon some viral infections (85), indicating functional relevance of these links.

To further explore these observations, we checked the correlation between the identified TFs and transcripts in a dataset of lung epithelial Calu-3 cells, where the expression levels of SPI1 and RUNX3 were relatively low but TTF2 presented a strong positive correlation to the inflammatory markers and to NEAT1, and a strong negative correlation to DANCR (**Figure 4D**). To conclude, RUNX3 regulates DANCR, and possibly its related inflammatory reaction in the lung; and SPI1 and TTF2 control its expression in the brain and in epithelial cells, respectively. Thus, DANCR and NEAT1’s regulation of the inflammatory markers appeared to be conducted by distinct TFs in a tissue- and context-specific manner and to be particularly prominent in the brain.

### Various Transcripts Connected to COVID-19 Severity Correlate With DANCR and NEAT1 Expression Through microRNA and Cholinergic Networks

**Figure 5:**
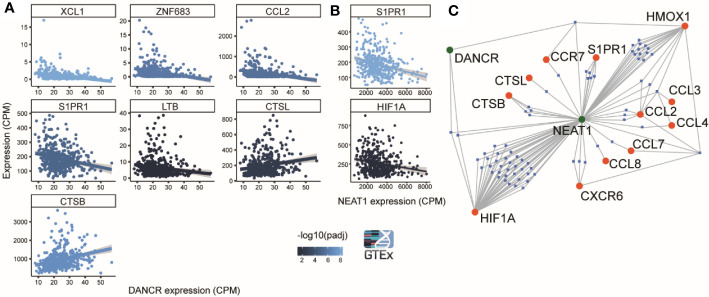
DANCR and NEAT1 expression networks are linked to genes discriminating between mild and severe SARS-CoV-2 insults. **(A)** 8 out of 19 genes found by Bost et al. (2020) to change between mild and sever SARS-CoV-2 infected patients show significantly correlated changes (p<0.05) to changes of DANCR (Pearson, FDR) in lung samples of 427 post-mortem GTEx donors. Gray region around the line represents SD for the linear fit. Line color represents significance [-log10(adjusted p value)] (86). **(B)** Same as A. for NEAT1. **(C)** A network of miRs (blue) that harbor binding sites on either DANCR or NEAT1 (green) and may target one or more of the 12 inflammation-related genes [(86) (red)].

Recent reports show distinct transcriptomic profiles in lung tissues from patients with mild compared to severe SARS-CoV-2 infection (86). To seek putative connections between mild and severe clinical patterns to DANCR and NEAT1 expression, we tested lung transcriptomes of 427 GTEx donors with and without inflammation characteristics. Supporting the relevance of DANCR’s expression changes to the individual differences between patients, we found that the differences in DANCR’s lung expression levels directly correlated to 8 out of the 19 genes discriminating between patients with distinct symptoms severity (Pearson, FDR, p<0.05; **Figure 5A**). In contradistinction, NEAT1 levels showed no correlation to 17 of those genes, and negatively correlated with the other two (Pearson, FDR, p<0.05; **Figure 5B**). Since we and others have shown that DANCR and NEAT1 can act as “sponges” to mediate inflammation by blocking the activity of “sponged” miRs (32, 87), we further sought plausible connections between these two lncRNAs and the transcripts discriminating between mild and severe COVID-19 patients; this analysis revealed a network including 12 of the inflammation-related transcripts whose levels were changed between mild and severe patients, and highlighted three genes three immune function-related genes (HIF1a, CCR7 and TLR4), whose expression may be co-regulated by both NEAT1 and DANCR (**Figure 5C**; interactions conducted *via* miRNet 2.0). Of those, HIF1a is activated during hypoxia, and its mal-functioning is implicated in various systems including the lungs. Correspondingly, HIF1a is involved in ALI (Acute Lung Injury), an inflammatory lung pathology considered to represent mild ARDS, changes in which are characteristic of complicated COVID-19 cases (12, 40, 88). Moreover, HIF1a is known to be causally involved in the reaction to inflammatory lung contusion and is extensively associated with immune cell function, and with reaction to influenza and tuberculosis infections (89–93). Furthermore, DANCR has been shown to affect HIF1a’s stability in malignancy, suggesting that the mechanism controlling this interaction may likewise be operational in other contexts, including infection control (58). In comparison, CCR7 is central for lymphocyte and dendritic cell functioning, including migration to tissues with active infection (94–96), and HMOX1 affects immune functions by controlling Heme metabolism. HMOX1 is further involved in interferon regulation following stimulation of the innate immune receptors TLR3 and TLR4, and has been implicated in various viral infections (97–99). Thus, DANCR and NEAT1 both appear to act as kernels to a network of genes involved in inflammation and diverse infections, at least in the context of COVID-19.

**Figure 6:**
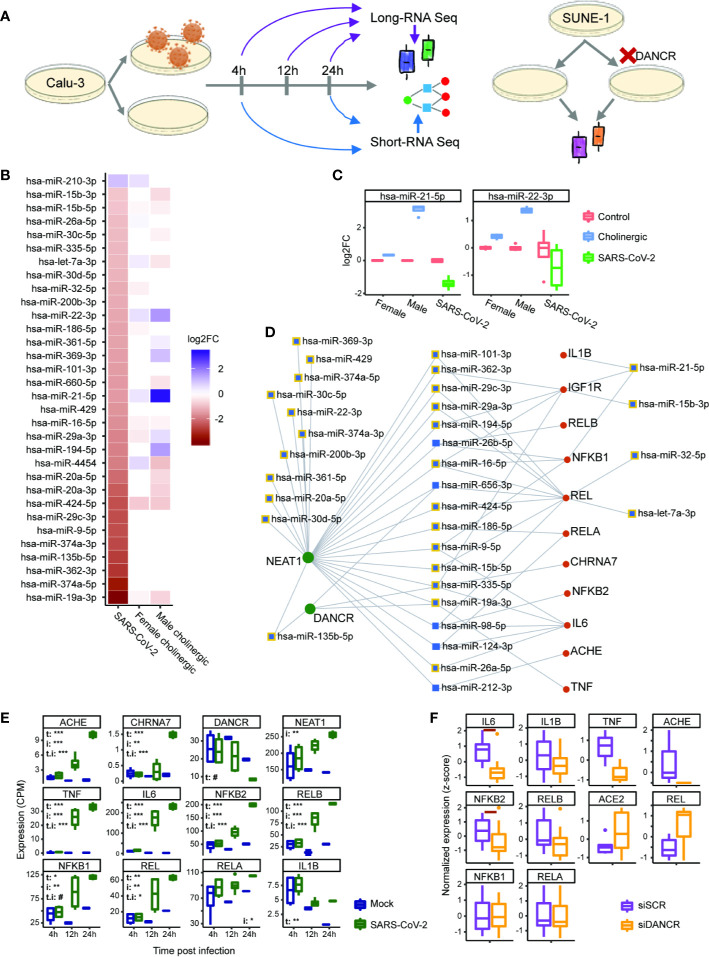
DANCR and NEAT1 interlink with inflammatory transcripts and DE miRs both in SARS-CoV-2 infection and in differentiated cholinergic neurons. **(A)** Long RNA-sequencing was conducted on Calu-3 epithelial lung cells infected or not with SARS-CoV-2 and harvested 4, 12, and 24 h post infection. Short RNA-sequencing was performed on 4- and 24-h samples. Additionally, neuron progenitor cells (SUNE-1) were treated with siDNACR (to execute DANCR knockdown) or with si-Scramble (siSCR) for control and were sequenced for long RNAs. **(B)** 32 miRs were DE in infected Calu-3 cells (left column) and were ordered from largest positive to negative fold change (FC; top; bottom). Thirteen and 17 of these miRs were DE under cholinergic differentiation in female and male neuroblastoma cells (LA-N-2; middle column and LA-N-5; right column). Color coding represents log2 of FC with red standing for negative FC of infected/uninfected or differentiated/non-differentiated and blue—for positive. n=4 for each condition. **(C)** Boxplot representation for two miRs from B. Red stands for all control conditions (untreated \ uninfected); blue—for cholinergic differentiation (CNTF) and green – for SARS-CoV-2 infection. Expression is normalized to the mean of control in each group (female, male and SARS-CoV-2-infection). **(D)** A network showing miRs (blue) targeting either DANCR or NEAT1 (green) and at least one inflammatory transcript (red). Squares representing miRs that were DE under SARS-CoV-2 infection are shown with yellow margins. **(E)** Boxplots showing Calu-3 infected (green) and control (blue) cells in three different timepoints. Upper-left corner shows significance for corrected (FDR) ANOVA test results (# = p<0.065; * = p<0.05; ** = p<0.01; *** = p<0.001) where t stands for “time” (i.e. 4, 12, or 24 h), i—for “infection” (infected or uninfected) and “t.i” for the combination of time and infection. If not stated—insignificant. n=3 for each condition. **(F)** SUNE-1 neuron progenitor cells treated with siDANCR (orange) or siSCR (purple). Red horizontal lines indicate p<0.05 (t. test, FDR). n=3 for each condition.

The expression of non-coding RNAs, including miRs, reflects characteristic cellular responses to inflammation and related cholinergic activity (32). Therefore, we postulated that miRs changed under SARS-CoV-2 infection will be co-affected also under cholinergic differentiation. To challenge this assumption, we examined miRs differentially expressed in lung epithelial cells (Calu-3) infected with SARS-CoV-2 in comparison to mock treated cells (4 or 24 h post-infection with MOI of 0.3). Calu-3 cell lines were used by others to challenge models of miRs whose levels are changed under infection with SARS-CoV-2 and the closely related SARS-CoV (76, 100–102). Strikingly, out of 32 differentially expressed (DE) miRs under SARS-CoV-2 infection, 21 were also DE in cholinergic neurons of either male or female lines following cholinergic differentiation (or both; **Figures 6A, B**). Of these miRs, particularly interesting are the pain-regulating miR-21-5p (103) and miR-22-3p, the levels of which presented opposite directions of change under cholinergic differentiation and SARS-CoV-2 infection, with male-originated cells showing a drastically stronger effect than female-originated ones (**Figure 6C**). Compatible with the ‘sponging’ property of both DANCR and NEAT1, the infection-related DE miRs showed intriguing interactions with DANCR, NEAT1, and the selected set of inflammation-related transcripts, eleven of which are targeted by 16 out of these 32 DE miRs; furthermore, both NEAT1 and DANCR carry complementary binding sites to 24 out of these 32 DE miRs, and 18 of the miRs targeted by NEAT1 and DANCR also target at least one of the tested inflammatory transcripts (**Figure 6D**). Specifically, miR-19a-3p and miR-335-5p target both NEAT1 and DANCR as well as couple of inflammation controlling transcripts and are downregulated under SARS-CoV-2 infection. Thus, the changes in DANCR and NEAT1 may lead to the infection-related impact of those miRs and regulate the inflammation signature associated with them in epithelial cells, albeit in a sex-related manner.

To challenge the possibility that the observed interactions are cell type-specific, we also explored long RNA-seq datasets in Calu-3 cells 4, 12, and 24 h post infection with SARS-CoV-2 and saw the same changes we found in NHBE cells. Namely, interleukins, NFkB-related genes, AChE and the α7 nicotinic receptor were all upregulated under SARS-CoV-2 infection, with their levels increasing with time (and the largest difference observed after 24 h). This was accompanied by DANCR downregulation after 24 h (p<0.067), contrasting the upregulation of NEAT1 levels in the infected Calu-3 cells (**Figure 6E**). Therefore, we next wished to re-challenge the connection between DANCR and the inflammation-related transcripts in other neuron types. To reach this goal, we examined a dataset of DANCR knockdown (KD) in neuron progenitor cells and found downregulation of cytokines and the non-canonical pathway NFkB (IL-6 and NFKB2, p<0.05, FDR; **Figure 6F**), indicating increased susceptibility to infection in differentiated neurons. Altogether, these multi-leveled experimental and analytic tests postulated that DANCR, NEAT1 and the cholinergic system’s transcripts are all pivotal inter-related factors in the reaction to SARS-CoV-2 infection in both lung cells and brain neurons.

## Discussion

Current COVID-19 diagnostics and treatment modalities are mainly utilized to detect and control its inflammation-related characteristics, but only partly address the inter-individual variability of patient populations. To seek predictive diagnostics and novel therapeutics for COVID-19 infection and its aftermath consequences, we pursued long and short ncRNAs susceptible to COVID-19 infection in lung, blood and brain cells by segregating 563 GTEx lung and blood samples into inflammation-prone and non-prone subjects, mining their RNA-seq datasets and studying cell culture viral infection, differentiation and genomic knockdown experiments. We discovered the lung pathology-associated lncRNA DANCR and the nuclear paraspeckles forming neuroprotective lncRNA NEAT1 as potentially involved in the susceptibility to and consequences of COVID-19, in conjunction with acetylcholine and inflammation-regulating transcripts. Notably, these observations were undetectable in blood samples, weakened with age and presented sex-dependent links in cholinergic neurons, highlighting lung cells as a preferable site for COVID-19 diagnostics. At the same token, these findings call for greater appreciation of the impact of medications with anti-cholinergic effects on post-infectious cognitive sequelae; these agents are commonly used as supportive measures in intubated and spontaneously breathing hospitalized patients. Taken together, our findings may assist future management of COVID-19 patients, as they may exhibit diverse expression profiles of molecular regulators such as DANCR and NEAT1, in conjunction with previously depicted inflammatory mediators such as IL-1, IL-6, TNF, and more. These two lncRNAs create a broad canvas for risk stratification, which needs to be further explored regarding short and long-term consequences of COVID-19. As the medical and scientific communities face the apparent neurologic sequelae of the hyper-inflammatory COVID-19, it is apparent that much is left yet uncharted; appreciation of the molecular mechanisms underlying this infection is therefore urgently needed, in order to highlight vulnerable populations, develop appropriate short and long-term follow up measures, and aid in devising therapeutic strategies.

By classifying lung transcriptomic data from apparently healthy GTEx donors to inflammation-prone or resilient, we found the expression of lncRNAs DANCR and NEAT1 to predict the inflammatory profile exhibited by involved tissues, and reflect on adverse body and brain consequences of COVID-19. Specifically, seeking lncRNA regulators of the susceptibility for COVID-19 identified the inflammation-modulating lncRNA DANCR as a potential chief regulator, responsible for the surveillance over cholinergic blockade of inflammation, and thus supporting the maintenance of a delicate balance between pro- and anti-inflammatory pathways in SARS-CoV-2-affected lung and brain tissues. The role of DANCR was evident in several model systems reflecting the reaction of lung epithelial cells to SARS-CoV-2 infectious challenge, with the timelines involved presenting an expected modulation of skewed inflammatory and cholinergic mediators, jointly orchestrated and balanced. The abovementioned connections of DANCR with STAT3 suggest a tentative feedback loop through which DANCR can affect the inflammatory response during infection. This pattern of surveillance is typically detected in inflammatory clinical entities, including lung infections (104). At the clinical-molecular interface, this balance emerged as being critical for coping with COVID-19-related pulmonary and systemic derangements as life-threatening ARDS and respiratory failure. The DANCR knockdown model as well supports the observed findings in COVID-19-related lung epithelial tissues, where DANCR emerges as an important inflammatory controller. Taken together, these findings extend the molecular inflammatory “thumbprint” reaction to include DANCR in conjunction with other previously established prognostic mediators in the context of COVID-19.

DANCR is notably active in lymphoid organs and T cell differentiation and presents massive increases following influenza vaccination (105, 106). Compatible with a regulatory role for DANCR in SARS-CoV-2-generated lung infection, DANCR shares a genomic locus with miR-4449, which is upregulated in airway epithelial cells under respiratory syncytial virus (RSV) infection (107), and is dysregulated in lymphocytes of multiple myeloma and ankylosing spondylitis patients (108, 109). Correspondingly, we identified DANCR as prominently reduced under SARS-CoV-2 infection, together with the lncRNA NEAT1 which controls RNA-regulating processes *via* forming the non-membranous organelle of the nuclear paraspeckles. Of note, NEAT1 interacts with inflammatory pathways, leading to transcriptional activation of IL-8 (110) and protecting human-originated neuronal cells under oxidative stress in a manner mediated by lipid lowering medications (such as statins) (33). NEAT1 is further implicated in inflammation by its association with controllers of inflammasome-associated proteins, and by its assembly with other molecular elements into an immune-controlling ribonuclear complex (111, 112). Our findings hence indicate that DANCR and NEAT1 may co-contribute to the inflammation-regulating ncRNA-mRNA network, operating in conjunction with other coding and non-coding mediators; this network’s interconnectivity may be appreciated when examining shared components such as miR-135b-5p, miR-656-3p, miR-335-5p, and miR-19a-3p, some putatively affecting inflammation by interacting with key related downstream mediators such as TNF and IL-6 or the nicotinic and muscarinic acetylcholine receptors.

Several of the miRs identified in our study have previously been implicated as influencing inflammatory pathways, such as ROCK1 and JAK1-STAT3, in the context of malignancy (113–117). The complex ncRNA-ncRNA interaction pattern, as described here, is apparent in other clinical contexts such as diabetes (itself a risk factor for adverse COVID-19 features) and may reflect this network’s inherent ability in these conditions. Furthermore, the cholinergic machinery is affected by shared ncRNA regulators (such as CHRNA7 affected by miR-335-5p (118)) which may be a molecular reflection of the inflammation cross-talk extensively described elsewhere (45, 119). Failure of these networks to mount an appropriate and balanced inflammatory reaction may therefore produce profound inflammation, inflicting extensive collateral damage as is indeed evident in COVID-19-related ARDS-like reactions. We conclude that the distinct clinical manifestations and characteristic individual variability in COVID-19 are underlined by ncRNA malfunctioning, leading to systemic maladaptation, skewed inflammation and “hyper-inflammatory” response.

Supporting a functional role of DANCR in COVID-19, we have further identified correlation of DANCR expression levels with those of the membrane-bound ACE2 protein in lung specimens of healthy donors. Although at this time the mechanisms underlying ACE2’s role in COVID-19 are incompletely understood, its apparent interaction with ncRNA regulators of the inflammatory machinery warrants further pursuit of its role in cellular derangement during infection. The joint involvement of ncRNAs in the cholinergic blockade of inflammation has been established in accumulating evidence thus far (45, 120–126). One example in the neurological context is the “sponging” activities of U1 ncRNA, which carries a complementary sequence to hsa-miR-125b-5p that has recently been shown to target AChE mRNA (56), and the levels of which are altered in brain cells from Alzheimer’s Disease patients (127). Taken together, our study suggests that cholinergic mRNAs and ncRNA regulators thereof may play a dominant role in both the severity of symptoms and the consequent neurological complications observed following COVID-19.

The lncRNA-mediated “overwatch” may avert cytokine storms characteristic of sepsis; and prevent detrimental life-threatening states which may develop where these and other mediators fail to harness overwhelming inflammation (35). Correspondingly, changes in inflammation-related ncRNAs, coupled with modified cholinergic surveillance, are evident in infectious, neuro-inflammatory and other conditions, including brain ramifications (30, 37, 119, 128–130). These include, for instance, the activity of the brain’s miR-218 in conjunction with cholinergic machinery in the context of amyotrophic lateral sclerosis (ALS) (131). MiR-218 was further shown to interact with the Slit2-Robo4 regulatory axis and TNF receptor 1 in other inflammation-intensive scenarios (132, 133), thereby raising the possibility of active neuro-inflammatory surveillance by this regulator.

Recent COVID-19 reports raise the importance of neurological complications inherent in the disease course (24, 134), indicating causal involvement of cholinergic-associated ncRNAs in CNS pathology (e.g. the AChE-targeting and inflammation-regulating miR-132 (45)). Furthermore, aged patients under prolonged anti-cholinergic medications are at increased risk of cognitive decline (135), and our findings of the apparent cholinergic-DANCR-NEAT1 links raise the question if such patients would also display excessive susceptibility to failed ncRNA response and hence harsh cognitive consequences of COVID-19. These effects may be augmented by adverse mental derangement associated with intensive-care settings and mechanical ventilation, but the relatively common loss of smell and taste in reports so far (22) suggests that SARS-CoV-2 has inherent neurological effects. It is perhaps too soon to tell how neurological COVID-19 complications progress in survivors during long-term follow up, but some reports regarding related coronaviruses suggest that these might harbor long-lasting effects associated with neurodegenerative and neuroinflammatory sequelae (136). Considering the increasing prevalence of COVID-19, together with intensive care phases for a substantial cohort of patients, it is plausible to expect long-lasting neurological effects in many. It is too soon to predict which patients would suffer from debilitating neurological sequelae, but further exploration of the neurological effects of COVID-19 is warranted; understanding the main mechanisms and developing risk assessment and stratification tools may identify particularly prone patient populations.

Based on accumulating clinical experience coupled with our current findings, we predict major ramifications of COVID-19 for healthcare, rehabilitation and social support systems. Having identified inflammation-associated ncRNAs as potential regulators of COVID-19 and possibly its ARDS-related pathogenesis (34, 137), we postulate relevance both for the acute phase, when caregivers seek specific acute management modalities, and for post-acute care for months and years following. Thus, as is generally noted in intensive care settings, the acute inflammation profile inherent to COVID-19 might expose both elderly and vulnerable young populations to variable risks of cognitive dysfunction post-intensive care (138), and ncRNAs may have pivotal roles in these inflammation-driven acute and long-lasting phases of COVID-19.

Other inflammatory phenomena inherent in viral infections such as influenza and COVID-19 include myocarditis, with clinical presentation ranging from mildly symptomatic to fulminant cardiac decompensation requiring inotropic support (139, 140). Compatible with the anti-inflammatory role of cholinergic routes are the known links between lung and brain injury. ARDS occurs in up to 25% of patients with severe brain injury, and its occurrence harbors poorer prognosis in these patients (18). Animal experiments reflect these interlinks at the molecular level, as intracranial hypertension augments the inflammatory response in ARDS, characterized by increased levels of TNFα, IL-6 and IL-1β, and microglial activation (141, 142). In cortical brain neurons, these neurokines target the cholinergic network (56), modulating the cholinergic blockade of inflammation. Age-related and sex-dependent decline of the cholinergic tone (143) may hence worsen the prospects of cognitive recovery in severely infected patients. The variability of clinical presentation of myocarditis, and the difficulty of diagnosis in resource-limited environments, may lead to under-diagnosis of such complications in these patients. Nevertheless, our view of COVID-19 as inflammation-intensive may predict potent extra-pulmonary cardiac and CNS complications (144).

Last, but not least, our findings may be relevant for assessing high-risk populations susceptible to adverse consequences of COVID-19, including obese (145, 146), hypertensive (4, 10, 12), and diabetic patients (13). Shared inflammatory machinery, activated by acute infectious entities and exacerbated by chronic comorbidities with active inflammation, may underlie these associations. Importantly, DANCR has been associated with diabetes mellitus *via* the cell metabolism regulator Insulin-like Growth Factor 2 mRNA Binding Protein 2 (IGF2BP2), which regulates DANCR’s expression and stability (147); additionally, DANCR regulates the PI3K/AKT pathway, which affects insulin homeostasis, and is also involved in inflammatory and cholinergic function (148–150). Thus, the non-coding regulators igniting and propagating COVID-19 may provide potential mechanistic explanations to the hazards incurred by at-risk populations, and our findings bring ncRNAs at large, and DANCR and NEAT1 in particular, to the realm of COVID-19 and its aftermath. Further suggestive of the inter-individual risk disparity in COVID-19 is the observed differential expression by sex of several miRs including miR-21-5p, which has been previously observed to be dysregulated and associated with immune function in infectious scenarios (151–154). In the current study, we also noted sex-related changes in the activity of cholinergic and inflammatory elements, inversely correlated to DANCR’s expression levels. Although at this stage it is difficult to ascertain how these differences explain the sex disparity in the consequences of COVID-19, it highlights DANCR as a putative marker for ongoing research in this question (155–158). Moreover, in the broad sense, identification of susceptible populations to COVID-19 mostly relies upon acute clinicopathologic features such as oxygen saturation levels and laboratory assessment of systemic inflammation, accompanied by patients’ background features such as age and comorbidities. Molecular aspects may aid in refining risk assessment in COVID-19, such as suggested in a recent case series of patients who had loss-of-function variants of TLR7, associated with impaired type I and II interferon responses (159). This further suggests that the molecular machinery propelling inflammatory responses, with its inter-individual variability, may play a dominant role in COVID-19.

Emerging therapeutics may operate in the space of inflammation generated by COVID-19; an example for such recently investigated agent, Remdesivir (160, 161) affects the stimulator of interferon genes (STING), itself implicated in regulating innate immune function. Likewise, ncRNAs such as miR-576-3p have been shown to be involved in STING’s operation (162, 163). Another investigated agent undergoing trials for COVID-19, ABX-464 (Abivax SA, clinical trial identifier NCT04393038), has been shown to upregulate the expression of miR-124 in other infectious contexts (164). Cholinergic signaling has also been found to regulate miR-124, with corresponding effects in inflammation signature pathways of STAT3, IL-6 and TNFa (122). It is therefore possible that these small regulatory molecules and their interactions with long non-coding RNAs are part of the COVID-19 pathogenesis. If so, they also provide therapeutic targets and opportunities for intervention, possibly in conjunction with other immunomodulatory efforts (165) and with agents which may upregulate the expression of NEAT1, such as statins (33). In conclusion, establishing the interlinks between specific ncRNAs and their targets may augment our understanding of the inflammatory course of COVID-19, aid in designing intervention strategies, and lead to potential novel diagnostic and therapeutic means.

## Data Availability Statement

All datasets presented in this study are included in the article/**Supplementary Material**.

## Ethics Statement

The studies involving human participants were reviewed and approved by The Genotype-Tissue Expression (GTEx) Project. The patients/participants provided their written informed consent to participate in this study.

## Author Contributions

CM led the clinical aspects of this study and wrote the corresponding parts in the manuscript, NM performed the experimental and analysis work and wrote the corresponding parts of the manuscript, and HS led and guided the project, including writing and editing of this manuscript. All authors contributed to the article and approved the submitted version.

## Funding

This study was supported by the Common Fund of the Office of the Director of the National Institutes of Health, and by NCI, NHGRI, NHLBI, NIDA, NIMH, and NINDS. Additionally, this study was supported by the European Research Council Advanced Award 321501, the Israel Science Foundation grant 1016/18, and the Israeli Ministry of Science, Technology and Space, Grant No. 53140 (to HS).

## Conflict of Interest

CM reports consultation fees from Raziel Therapeutics ltd., outside of the submitted work. HS reports research support from Grunenthal, Ltd. on pain-related miRs, outside of the submitted work.

The remaining author declares that the research was conducted in the absence of any commercial or financial relationships that could be construed as a potential conflict of interest.

## Supplementary Material

Supplementary Table 1 lncRNAs expressed distinctly in infected and non-infected SARS-CoV-2 NHBE and A549 cells, pandemic H1N1 NHBE cells, and differentiating between inflammation-prone (n=57) and non-prone (n=362) lung samples from GTEx individuals.

Supplementary Table 2 miRs with predicted inflammatory targets whose levels were changed during cholinergic differentiation of LA-N-2 female and LA-N-5 male neuroblastoma cells. Those were extracted by running DESeq2 analysis (R studio) on the nondifferentiated and differentiated cultured cells.

## Abbreviations

ACE2, Angiotensin I Converting Enzyme 2; ACh, Acetylcholine; AChE, Acetylcholinesterase; ALI, Acute Lung Injury; ALS, Amyotrophic Lateral Sclerosis; ARDS, Acute Respiratory Distress Syndrome; ChAT, Choline-acetyl Transferase; CNS, Central Nervous System; DE, Differentially Expressed; IL, Interleukin; lncRNA, long non-coding RNA; MERS, Middle-East Respiratory Syndrome; miR, microRNA; MOI, Multiplicity of Infection; mRNA, messenger RNA; ncRNA, non-coding RNA; RNA-seq, RNA sequencing; SARS, Severe Acute Respiratory Syndrome; TF – Transcription Factor; TNF, Tumor Necrosis Factor; VAChT, Vesicular Acetylcholine Transporter.
