# Supplementary material for: The Neat Dance of COVID-19: NEAT1, DANCR, and Co-Modulated Cholinergic RNAs Link to Inflammation
Source: Front Immunol. 2020 Oct 9;11:590870. doi: 10.3389/fimmu.2020.590870 (PMC7581732; doi:10.3389/fimmu.2020.590870)
Supplement: Supplementary file 1 [file DataSheet_1.pdf]

Figure S1:

S1 Blood shows minimal DANCR difference with age

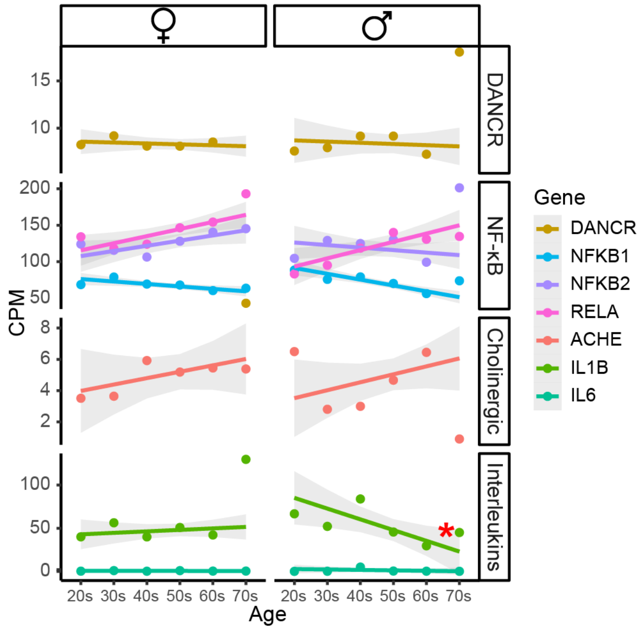

Correlation of various transcript levels with age in 400 blood samples. Red asterisks indicate significance of p<0.05 (FDR).
